# Antibiotic resistance in plant growth promoting bacteria: A comprehensive review and future perspectives to mitigate potential gene invasion risks

**DOI:** 10.3389/fmicb.2022.999988

**Published:** 2022-09-20

**Authors:** Ismail Mahdi, Nidal Fahsi, Mohamed Hijri, Mansour Sobeh

**Affiliations:** ^1^Agrobiosciences Research Program, Mohammed VI Polytechnic University (UM6P), Ben Guerir, Morocco; ^2^Institut de Recherche en Biologie Végétale, Département de Sciences Biologiques, Université de Montréal, Montréal, QC, Canada; ^3^African Genome Center, Mohammed VI Polytechnic University (UM6P), Ben Guerir, Morocco

**Keywords:** antibiotic resistance genes (ARGs), biofertilizers, gene invasion risks, plant growth promoting bacteria (PGPB), public health

## Abstract

Plant growth-promoting bacteria (PGPB) are endowed with several attributes that can be beneficial for host plants. They opened myriad doors toward green technology approach to reduce the use of chemical inputs, improve soil fertility, and promote plants’ health. However, many of these PGPB harbor antibiotic resistance genes (ARGs). Less attention has been given to multi-resistant bacterial bioinoculants which may transfer their ARGs to native soil microbial communities and other environmental reservoirs including animals, waters, and humans. Therefore, large-scale inoculation of crops by ARGs-harboring bacteria could worsen the evolution and dissemination of antibiotic resistance and aggravate the negative impacts on such ecosystem and ultimately public health. Their introduction into the soil could serve as ARGs invasion which may inter into the food chain. In this review, we underscore the antibiotic resistance of plant-associated bacteria, criticize the lack of consideration for this phenomenon in the screening and application processes, and provide some recommendations as well as a regulation framework relating to the development of bacteria-based biofertilizers to aid maximizing their value and applications in crop improvement while reducing the risks of ARGs invasion.

## General introduction

In the last few years, there has been growing interest in using microbial-based products as bioinoculants or/and substances. However, their use is facing many challenges from the lab to the field (Madawala, 2021). Plant growth-promoting bacteria (PGPB) are a polyphyletic group of bacteria that interact closely or loosely with plant roots in the rhizosphere biotope. These bacteria have direct or indirect positive effects on plant growth and health. According to Klopper (1980), PGPB are distinguished by their ability to colonize the roots, survive, multiply, and become competitive with other microorganisms (Klopper, 1980). They can increase the availability of nutrients, regulate the production of phytohormones, increase tolerance to abiotic stresses and inhibit pests and diseases by competition (Glick, 2012; Souza et al., 2015).

The intensive and continuous use of chemical fertilizers and pesticides in the last 50 years has substantially influenced public health and agricultural productivity in the world (Ramakrishna et al., 2019). However, these practices have resulted in multiple environmental concerns mainly soil fertility degradation, reduced biodiversity in soils, increased greenhouse gas emission, zinc deficiency, chemical residues accumulation, and eutrophication among others (Tilman et al., 2002; Diaz and Rosenberg, 2008). Over the last few decades, more eco-friendly approaches such as the use of PGPB as biofertilizers have gained popularity in moving toward a sustainable agroecosystem (Ramakrishna et al., 2019). On the economic perspective, biofertilizers market shows a growth of more than 12% increase *per annum* (Calvo et al., 2014; Owen et al., 2015). The largest market represented by Nitrogen-based biofertilizers was estimated to grow by up to 13.25% per year by 2020 (Arora, 2018). To date, many PGPB strains belonging to the genera of *Acinetobacter*, *Agrobacterium*, *Azospirillum*, *Bacillus*, *Bradyrhizobium*, *Burkholderia*, *Enterobacter*, *Gluconacetobacter*, *Pantoae*, *Pseudomonas*, *Rhodococcus*, and *Serratia* have been reported to have beneficial effects on plant growth promotion, nutrition, stress alleviation, bioremediation, and/or biocontrol of plant pathogen’s attacks (Sharma et al., 2013; Bargaz et al., 2018). Large-scale production and commercialization of bacterial-based biofertilizers have been achieved using some PGPB belonging to the genera of *Azospirillum*, *Azotobacter, Bacillus*, *Burkholderia*, *Pseudomonas* and *Rhizobium* (Parray et al., 2016). Despite the obvious agronomic advantages that biofertilization offers, some earlier studies have raised some concerns that could lead to negative effects of their application on the behavior and genetic profile of soil microbial communities and have sounded the alarm about their intensive and unreasonable long-term application (Lee et al., 2018). For instance, the proximity and the high microbial density (indigenous or introduced) in the rhizosphere were reported to enhance the horizontal (or lateral) gene transfer (HGT) among bacterial species. HGT is known to be one of the major mechanisms in bacterial evolution (Popa and Dagan, 2011). These transferable genetic elements could harbor ARGs and virulence factors that could pose human health risks. In this context, this review focuses on the paradox behind the use of antibiotic-resistant PGPB, addresses potential risks for public health, and provides a regulation framework with practical solutions and recommendations as well as the approaches of isolation and development of effective and biosafe PGPB-based biofertilizers.

**Figure 1:**
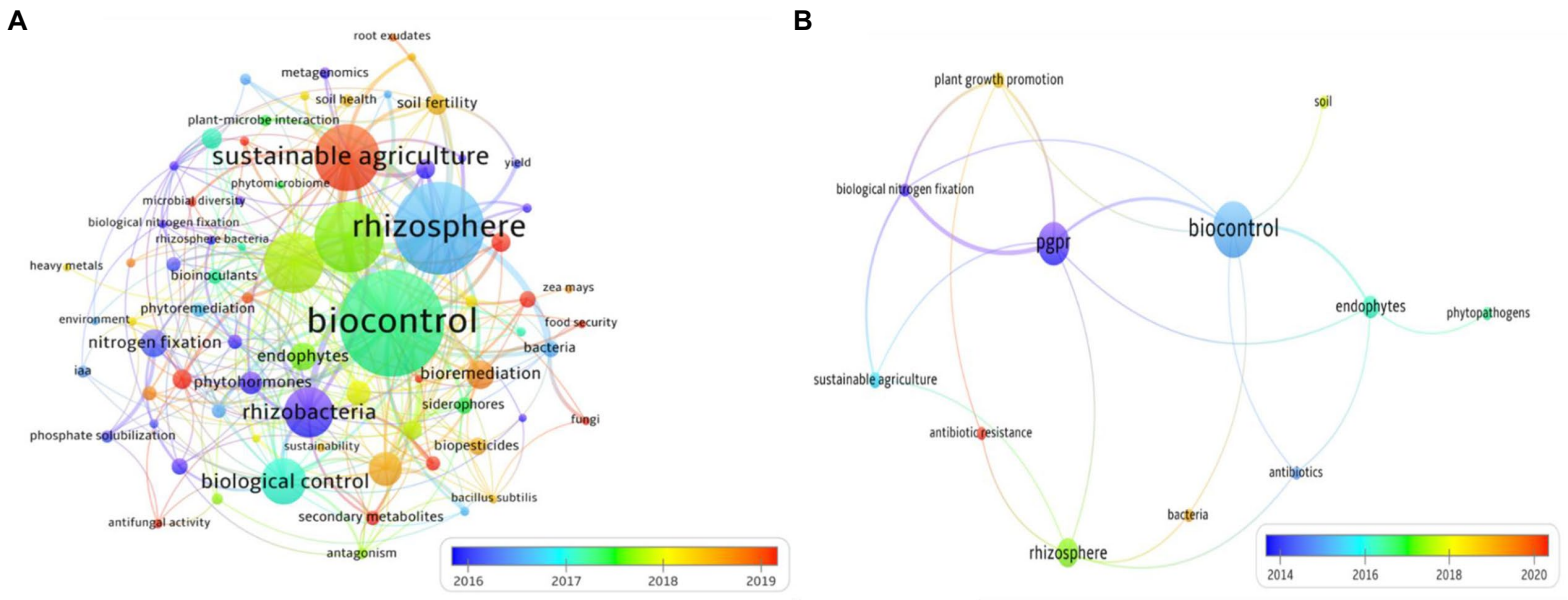
**(A)** Co-word map network visualization and **(B)** Knowledge map of antibiotic resistance in bacterial-based biofertilizers. The larger the circles are, the more scientific publications were found. PGPR, plant growth promoting rhizobacteria.

Data analyzed in this review were collected using the Scopus research engine. The search was launched by entering the keywords “PGPB,” “Endophyte” and refining with additional keywords “antibiotic,” “resistance,” and “antibiotic resistance genes.” A total of 398 hits were retained for further analyses in the scope of this review. Therefore, we used the co-occurrence mapping tool of relevant keywords which resulted in more than 50 high frequency keywords that were used to construct a knowledge map of the main strong domains of research studies related to PGPB and resistance to antibiotics. Noteworthy, when the terms “antibiotic AND resistance” were absent, records increased to 21,199 hits. Figure 1 shows the networks inferred using pertinent keywords and co-occurrence analysis in the dataset. Based on the relevant keywords, assigned clusters were clearly identified by the following keywords: “rhizosphere,” “rhizobacteria,” “sustainable agriculture” and “biocontrol” (Figure 1A). These four clusters are strongly connected to each other. As shown in Figure 1B, the main recent topic in the field of biological activities of biofertilizers is “antibiotic resistance” in which the number of publications has exploded since 2020 (Circled in red). This result shows that there is a need for more investigation into the extent of antibiotic resistance in PGPB. This feature is most likely due to the search by scientists for more persistent and competitive bioinoculants/biofertilizers but very rarely to their awareness about the danger that could be posed by PGPB species with a broad spectrum of antibiotic resistance.

## Plant-associated bacteria

**Figure 2:**
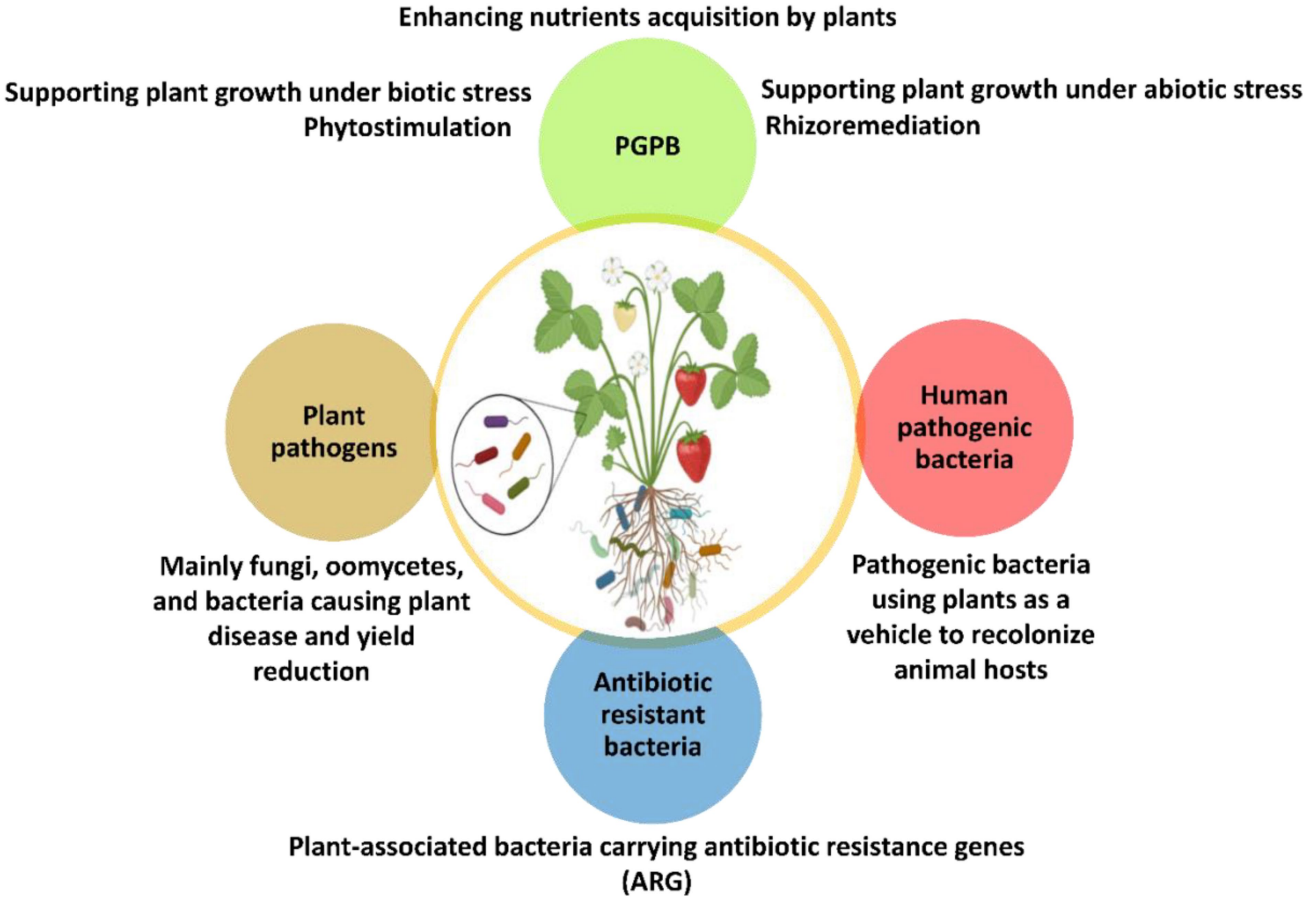
Schematic overview of plant-associated microorganisms and their respective functions. They live inside the plant tissues as endophytes or surrounding the root system as rhizosphere microorganisms. PGPB, Plant growth promoting bacteria.

While many soil bacteria are beneficial to plants, some are well known plant pathogens that cause severe damage and can lead to the complete losses of crop yields (Syed Ab Rahman et al., 2018). Such bacterial plant pathogens are able overcome the innate plant defense by different molecular mechanisms involving microbial effectors (Toruño et al., 2016). Another group of bacteria in the rhizosphere comprises opportunistic human pathogenic bacteria (HPB) which have a ubiquitous habitats including plant biotopes (Figure 2).

It is well established that bacteria can naturally integrate exogenous DNA including ARGs (Boto et al., 2019). Among the mechanisms by which bacteria acquire new functions are conjugation (transfer of mobile genetic elements by pili), transformation (uptake of DNA by competent bacteria), and transduction (transfer of DNA between a bacteriophage and a bacterium; Thomas and Nielsen, 2005; Arber, 2014). In addition, in some species, other mechanisms have been documented to help bacteria encapsulating DNA such as phage-derived sequences, and transfer mediated by vesicles produced in the outer membrane vesicles (García-Aljaro et al., 2017; Sun, 2018). Furthermore, many bacteria possess resistance elements located within the antibiotic biosynthetic cluster for self-protection and survival strategy (Hubbard and Walsh, 2003). This consolidates the fact that the intrinsic resistance is an ancient phenomenon that precedes the anthropogenic use of antibiotics, contradicting the common assumption that antibiotic resistance is uniquely related to antibiotic misuse (Cox and Wright, 2013). although, this does not exclude the impact of human activity in the resistance phenomenon and its dissemination. In fact, HGT can be promoted by anthropogenic activities by (i) favoring ecological forms of life such as biofilms where gene transfer can occur, (ii) inducing selective pressures that maintain transferred genes, and (iii) affecting HGT mechanisms through compounds such as legacy pollutants. Consequently, the potential threat of antibiotic resistance and HGT in affected environments such as plant–soil microcosm is worth inspecting (Andam et al., 2015; Gillings, 2017b).

Bacterial inoculants applied in large amounts for biofertilization, biocontrol, phytostimulation, or bioremediation of soils could increase HGT events with indigenous prokaryotes living in the soil and/or plant environments (Séveno et al., 2002). Several studies have addressed the emergence and propagation of ARGs in different environmental reservoirs through anthropogenic activities such as the application of manure, slurry and soil amendments with regard to their different transfer pathways and threats to human being health and ecology (Wellington et al., 2013).

**Figure 3:**
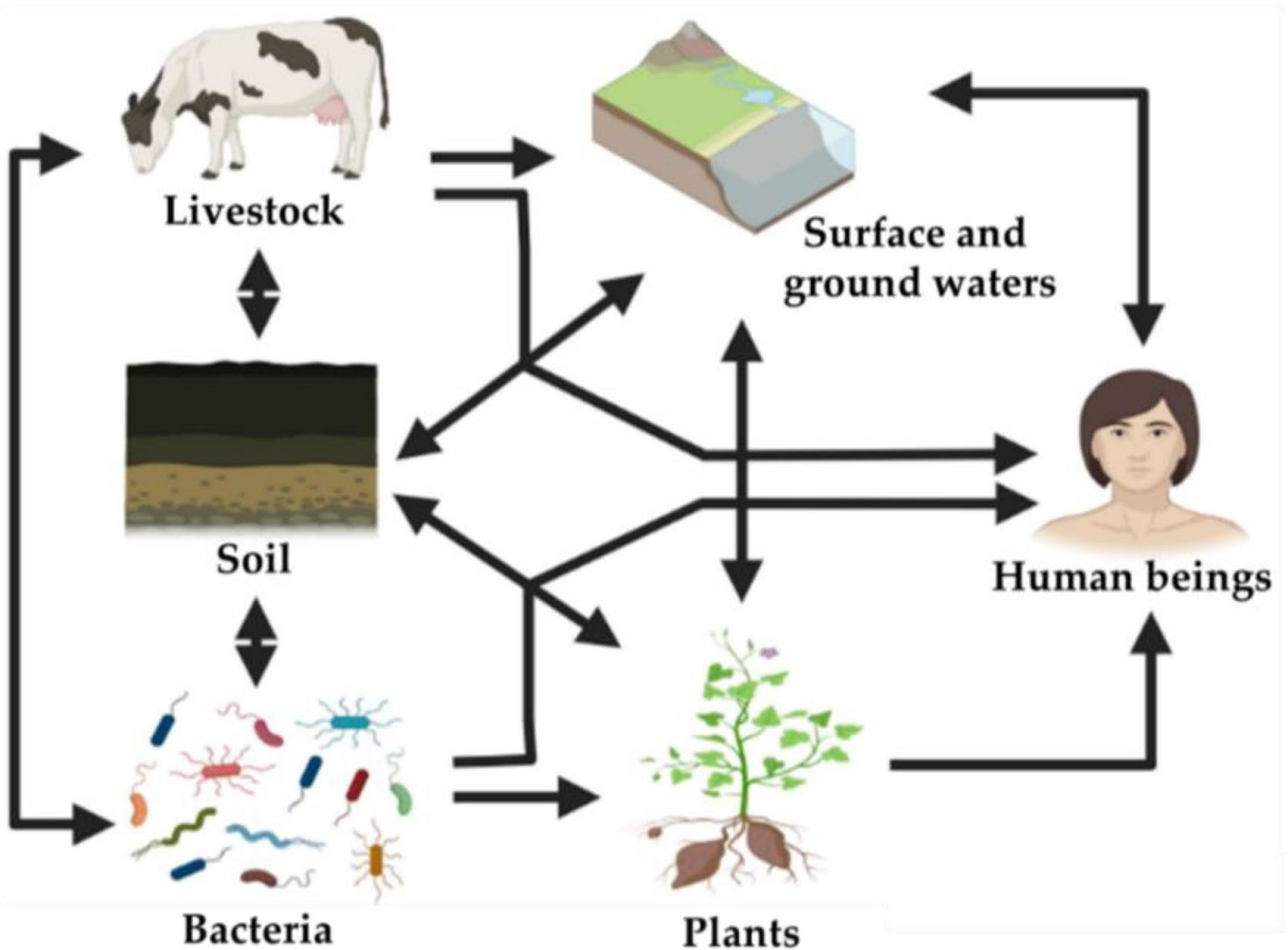
Schematic illustration of the associations between some key environmental settings of antibiotic resistant-bacteria and antibiotic resistance genes (ARGs). Acquisition of existing ARGs from the environmental microbiome occur mostly by horizontal gene transfer (HGT). Resistance may also occur in soil bacteria *via* mutations encoding target-site alterations.

In fact, the plant microbiome represents an interface between humans, microbes, and genes including those involved in the antibiotic resistance. Even nonculturable bacteria in soils remain metabolically active and retain virulence factors capable of causing infections in plants, animals and humans (Del Mar Lleò et al., 2007; Fakruddin et al., 2013). In suitable conditions, these bacteria could cause severe infections and diseases after ingestion as they are able to recover their metabolic activities (Baffone et al., 2003; Rowan, 2004; Epstein, 2009). Moreover, nonculturable bacteria are considered as a reservoir of pathogens in the environment. They include many human pathogenic bacteria such as *Enterobacter cloacae*, *Pseudomonas aeruginosa*, and *Shigella* strains (Fakruddin et al., 2013). Consequently, plant microbiome could indirectly influence the composition and function of the human microbiome resulting to potential human health issues (Chen et al., 2019). An analogy can be drawn with plant–soil microbiome feedback loop. In turn, humans could contribute directly or indirectly to the invasion of ARGs in plant microbiomes. However, PGPB harboring ARGs and bacterial biological control agents are mostly unexplored, while most of these PGPB are still recommended as effective biofertilizers (Supplementary Figure S1). Despite the importance of antibiotic resistance that benefits PGPB to survive within the indigenous microbiota in antibiotic contaminated areas (Cray et al., 2013), large-scale use of ARGs-harboring biofertilizers in the soil could spread antibiotic resistance in other ecosystems (Figures 2, 3). This concern about emerging antibiotic resistant bacteria is a double-edged sword in both sustainable agriculture and public health associated risks.

## Overview of mechanisms utilized by PGPB

Many mechanisms by which beneficial bacteria enhance nutrients acquisition by plants, are well documented in the literature (Vejan et al., 2016). The most common mechanism is changes of soil pH through bacterial organic acids secretion and proton release *via* stimulation of ATPase proton pump, which decreases soil pH and increases the concentration of soluble nutrients such as phosphorus and potassium (Mantelin and Touraine, 2004). Another example is the modulation of phytohormone biosynthesis through the production of various extracellular molecules (i.e., siderophores) and enzymes such as aminocyclopropane-1-carboxylate deaminase, which decrease ethylene levels in plants, thus facilitating plant growth and development and mitigating abiotic stress (i.e., salinity; Nadeem et al., 2007; Zahir et al., 2008). Other mechanisms involved in the alleviation of abiotic stresses in plants have include the production of plant hormones (auxins, gibberellins, and cytokinins), exopolysaccharides, osmoprotectors, and antioxidant enzymes (Egamberdieva et al., 2019).

Nitrogen fixation is mediated by soil bacteria which can form nodules in the roots, allowing to produce ammonia which contributes the plant nutrition (Murray, 2011). Furthermore, some PGPB protect plants against attacks of pests and pathogens (Glick, 2012; Ahemad and Kibret, 2014). This biocontrol activities are due to their ability to biosynthesize antimicrobial metabolites, such as hydrogen cyanide (HNC), phenazines, pyoluteorin, 2,4-diacetylphloroglucinol, tensin, viscosinamide, and pyrrolnitrin, etc.; to compete against the pathogens for nutrients; and to stimulate plant immune system (Plattner and Soldati-Favre, 2008; Planchamp et al., 2015). In addition, the priming effect of PGPB through volatiles organic compounds 2,3-butanediol, has emerged recently to account for biocontrol effects (Farag et al., 2013) against some fungal pathogens (Supplementary Figure S1).

## Contribution of PGPB on putative invasion and dispersal of antibiotic resistance genes

To become a successful PGPB-based inoculant, the candidate bacterial strains should demonstrate not only plant growth promotion traits, but also the ability for cost-effective mass production and formulation with a long shelf life (Nakkeeran et al., 2005; Tabassum et al., 2017). Although several PGPB have been reported to be carrying ARGs (Table 1), the extent of this threat and the potential for resistance transmission to soil microorganisms and ultimately its impact on human-associated bacteria is mostly underexplored. Antibiotics exert natural selection pressure on bacterial populations which often lead to increase the abundance of resistant bacteria in the environment. This major public health problem is strongly linked to the misuse and overconsumption of antibiotics in human and animal health. Consequently, the upsurge in multi-resistant bacterial strains resulted in substantially decreased of treatments success, thus causing increased bacterial infections potentially leading to mortality (Hawkey and Jones, 2009; Paul et al., 2010).

**Table 1:** A list of antibiotic resistance patterns of some declared PGPB strains and respective/putative identified ARGs.

Genus	Species	Isolation source/Host	Antibiotic resistance profile	Antibiotic resistance genes	References
*Bacillus*	*B. cereus*	Rhizosphere of sunflower	Hygromycin B, Chloramphenicol, Streptomycin, Penicillin, Fosfomycin, Bacitracin, Teicoplanin, and Vancomycin	bl2a; bacA; bcrA; fosB; and vanSA	Ivanova et al., 2003; Joo et al., 2004; Khan et al., 2018
	*Bacillus* sp.	Rhizosphere of Jujube	Ampicillin	ND	Fahsi et al., 2020
	*B. amyloliquefaciens* FZB42	Commercial	Tetracycline, Fosfomycin, Bacitracin, and Lincomycin	mdr, fosB, lmrB, ykkD, ykkC, bacA, and σW target genes	Chen et al., 2007; Idris et al., 2007
	*B. xiamenensis* PM14	Rhizosphere of sugarcane	Ampicillin, Kanamycin, Erythromycin, Chloramphenicol, Gentamycin, Fosfomycine, Spectinomycin, Lincomycin, Rifampicin, Penicillin, Streptomycin, and Clindamycin	ND	Amna Xia et al., 2020
	*B. licheniformis* and *B. paralicheniformis*	Soil, Plants, Environment	Not given	cat, aph, aadK, and ermD	Agersø et al., 2019
	*B. licheniformis* DSM 13	Commercial Roots of maize	Fosfomycin, Bacitracin, Lincosamide, Penicillin, Macrolide, and Streptogramin B	fosB, ermD, bacA, and bl2a	Gutiérrez-Mañero et al., 2001; Rey et al., 2004
	*B. cereus* ATCC 14579	Commercial Agricultural fields	Tertracycline, Penicillin, Vancomycin, Fosfomycin, Bacitracin, and Teicoplanin	fosB, vanSA, bl2a, bacA, bcrA, and tet	Ivanova et al., 2003; Joo et al., 2004
	*B. subtilis* 168	Commercial Bean plants	Tetracycline, Streptogramin B, Chloramphenicol, Fluoroquinolone, Doxorubicin, Puromycin, Lincomycin, Tunicamycin, Bacitracin, Fosfomycin, Lincosamide, and Macrolide	tetL, blt, bacA, ykkD, lmrB, mdr, ykkC, tmrB, bmr, fosB, and mls	Monod et al., 1986; Butcher and Helmann, 2006; Barbe et al., 2009
	*Bacillus* sp. RM-2	Rhizosphere of mung bean	Cephalxin, nalidixic acid	ND	Minaxi Nain et al., 2012
	*B. simplex* SH-B26	Rhizosphere of sugar beet	Tetracycline, Quinolone, Fosmidomycin, and Bacitracin	fosB, bacA, and σW target genes	Gutiérrez-Luna et al., 2010; Khayi et al., 2015
	*B. thuringiensis* 97–27	Soya bean root tissue	Penicillin, Bacitracin, Fosfomycin, Vancomycin, and Teicoplanin	fosB, vanRB, bl2a, bacA, and vanSA	Han et al., 2006; Calvo et al., 2010
	*Paenibacillus polymyxa* SC2	Rhizosphere of pepper	Tetracycline, Quinolone, Daunorubicin, Teicoplanin, Bacitracin, Lincomycin, and Fosmidomycin	lmrB, bacA, drrA, vanZ, and norA	Ma et al., 2011
	*Paenibacillus pabuli*	Sweet potato rhizosphere	Chloramphenicol, Streptomycin, Kanamycin, Penicillin, and Tetracyclin	ND	Yasmin et al., 2009
	*Paenibacillus xylanexedens* J155	Rhizosphere of Jujube	Streptomycin, Kanamycin	ND	Fahsi et al., 2021
*Pseudomonas*	*P. aeruginosa*	Rhizosphere of Vigna radiata (mung bean)	Nalidixic acid, Ampicillin, Chloramphenicol, Tetracycline, Erythromycin, and Cotrimoxazole	ND	Bakthavatchalu et al., 2012; Kumari et al., 2018
	*Pseudomonas* sp.	Rhizosphere of Jujube and Quinoa	Ampicillin, Spectinomycin, and Chloramphenicol	ND	Fahsi et al., 2020; Mahdi et al., 2021a
	*P. libanensis*	Rhizosphere of *Phleum phleoides*	Ampicillin, Tetracycline, Streptomycin, Chloramphenicol, and Penicillin	ND	Ma et al., 2016b
	*P. reactans*	rhizosphere of *Trifolium repens*	Ampicillin, Tetracycline, Streptomycin, Chloramphenicol, and Penicillin	ND	Ma et al., 2016b
	*P. pseudoalcaligenes* strain CECT 5344	Mineral soil samples	Beta-lactam, Novobiocin	mdtA, mdtB, mdtC, and bl3VIM	Luque-Almagro et al., 2013; Jha and Subramanian, 2014
	*Pseudomonas* sp. SH-C52	Rhizosphere of sugar beet	ND	thaABC1C2, braABCDE, and tnpABC	Paterson et al., 2016
	*P. chlororaphis* GP72	Green pepper rhizosphere	Chloramphenicol, Bleomycin	emrB, rarD	Ma et al., 2011; Kang et al., 2013
	*P. stutzeri* A1501	Rice paddies Maize rhizosphere	Gentamicin, Bacitracin, Dibekacin, Apramycin, Sisomicin, Tobramycin, and Netilmicin	bacA, aac(3)IV, and mexW	Ma et al., 2011
	*P. putida* KT2440	Maize rhizosphere	Chloramphenicol, Bacitracin, Erythromycin, Fluoroquinolone, Roxithromycin, and Glycylcycline	mexF, mexD, bacA, and mexW	Akhtar and Siddiqui, 2008; Luque-Almagro et al., 2013)
	*P. aeruginosa* PAO1	Rhizosphere soil of castor plants Mung bean rhizosphere	Chloramphenicol, Bacitracin, Erythromycin, Tetracycline, Fluoroquinolone, Beta-lactamines, Aminoglycoside, Glycylcycline, Cephalosporin, Roxithromycin, and Tigecycline	oprJ, oprM, oprN, catB4, mexA, mexB, mexD, mexE, mexF, mexI, mexW, mexX, mexY, mexH, mexY, oprN, bl1, bacA, opmD, aadA1, aafA5, blaVIM-2, tetA, tetR, and sul1	Salunkhe et al., 2005; Kumar et al., 2009
	*P. protegens* Pf-5 (Known as *P. fluorescens* Pf-5)	Commercial Populus deltoides tree	Chloramphenicol, Bacitracin, Erythromycin, Fluoroquinolone, Roxithromycin, and Glycylcycline	bl1, bacA, mexD, mexF, and mexW	Paulsen et al., 2005; Jha and Subramanian, 2014
	*P. fuscovaginae*	Sweet potato rhizosphere	Chloramphenicol, Streptomycin, Kanamycin, Penicillin, and Tetracycline	ND	Yasmin et al., 2009
	*P. maculicola*	Sweet potato rhizosphere	Chloramphenicol, Streptomycin, Kanamycin, Penicillin, and Tetracycline	ND	Yasmin et al., 2009
	*P. corrugate*	Sweet potato rhizosphere	Chloramphenicol, Streptomycin, Kanamycin, Penicillin, and Tetracycline	ND	Yasmin et al., 2009
	*P. frederiksbergensis* S6	Rhizosphere of quinoa	Ampicillin, Chloramphenicol	ND	Mahdi et al., 2021a
	*P. moraviensis*	Rhizosphere of Jujube	Chloramphenicol, Streptomycin, Kanamycin, Ampicillin	ND	Fahsi et al., 2021
*Serratia*	*S. marcescens* FGI94	Leaf-cutter ant fungus gardens	Spectinomycin, Gentamincin, Tetracycline, Streptomycin, Chloramphenicol, Kanamycin, Fosfomycin, Neomycin, Nalidixic acid, Bacitracin, Beta-lactamines, and Amikacin, Cephalosporin, Dibekacin, Tobramycin, Isepamicin, Polymyxin, Kasugamycin, Sisomicin, Netilmicin, Lividomycin, Paromomycin, Ribostamycin, Novobiocin, Macrolide, Bicyclomycin, and Trimethoprim	aph3IA, catB3, aac6IB, fosA, bl1SM, bl2F, bl2B, ksgA, acrD, bl3, dfrA1, marC, mexE, emrA, bacA, tolC, arnA, mdtA, mdtC, mdtB, ant3IA, tet41, and tetB	Aylward et al., 2013
	*S. fonticola* DSM 4576	Pea rhizosphere Sesame rhizosphere Rye soil	Novobiocin, Nalidixic acid, Bacitracin, Beta-lactamines, Fluoroquinolone, Fosmidomycin, Norfloxacin, Bleomycin, Fosfomycin, Vancomycin, Enoxacin, Bicyclomycin, and Polymyxin	bacA, bl2BE, mdtA, mdtB, mdtC, marC, mdtH, and emrA	Lim et al., 2015
	*S. ficaria*	Sweet potato rhizosphere	Chloramphenicol, Streptomycin, Kanamycin, Penicillin, and Tetracycline	ND	Yasmin et al., 2009
	*S. rubidaea* ED1	Roots of Quinoa	Ciprofloxacin, Meropenem, Ertapenem, Ampicillin, Chloramphenicol, Spectinomycin, and Tetracycline	ND	Mahdi et al., 2021b
	*S. plymuthica* MBSA-MJ1	Poplar trees, Oilseed rape, wheat, pumpkin, potato, maize, rice	Chloramphenicol, tetracycline	Cat, stp, bcr, fsr, arnABCD, ampC, ampR, ampD	Nordstedt and Jones, 2021
*Burkholderia*	*B. cepacia* RRE25	Rhizosphere of a wild plant Parthenium Hysterophorus	Tetracycline, Nalidixic acid, Bleomycin, Penicillin, Fosmidomycin, Aminoglycoside, Chloramphenicol, Novobiocin, Teicoplanin, Bleomycin, Dibekacin, Tobramycin, Cloxacillin, Isepamicin, Netilmicin, Amikacin, and Sisomicin	aac6IB, amrA, ceoA, ceoB, bl2D, emrA, marC, mexX, mdtC, mdtB, mdtA, tetA, and vanZ	Singh and Cameotra, 2013
	*B. phytofirmans* PsJN	Potatoes, canola, maize, and grapevines	Chloramphenicol, Nalidixic acid, Novobiocin, Bicyclomycin, Fosmidomycin, Teicoplanin, Bacitracin, and Bleomycin	bacA, ceoB, emrA, emrB, mdtC, mdtB, mdtA, and vanZ	Weilharter et al., 2011
*Pantoea*	*P. ananatis* AMG521	Rice endophyte	Chloramphenicol, Polymyxin, Nalidixic acid, Bacitracin, Novobiocin, Fosfomycin, Sulfathiazole, Kasugamycin, and Fosmidomycin	arnA, amA, bacA, emrD, emrB, emrA, marA, mdtC, mdtB, mdtA, mdtI, mdtJ, marA, ksgA, rarD, and tolC	Hoon et al., 2007; Megías et al., 2016
	*P. agglomerans* P10c	Commercial biocontrol strain	Chloramphenicol, Streptomycin, Polymyxin, Nalidixic acid, Penicillin, Bacitracin, Novobiocin, Fosfomycin, Bicyclomycin, Kasugamycin, Cloxacillin, Spectinomycin, and Fluoroquinolone	acrD, ant3IA, bacA, bl2BE, catB3, emrD, emrA, mdtC, mdtB, mdtA, ksgA, rarD, tolC, and qnrB	Smits et al., 2015
*Bradyrhizobium*	*B. japonicum* E109	Commercial rhizobacteria for soybean	Tetracycline, Fosmidomycin, Bicyclomycin, and Bacitracin	bacA, emrB, marC, qacA, and vanZ	Torres et al., 2015
	*B. diazoefficiens* USDA 110	Shrubby sophora (Sophora flavescens) and Soybean (Glycine max)	Tetracycline, Fosmidomycin, and Bacitracin	bacA, emrB, fsr, and tetA	Berquó Marks et al., 2013
	*Bradyrhizobium* sp.	Nodules borne on the root of Greengram plants	Kanamycin, Ampicillin, Tetracycline, Gentamycin, Streptomycin, Chloramphenicol, Nalidixic acid, and Trimethoprim	ND	Gupta et al., 1998
*Microbacterium*	*M. natoriense* strain E38	Rhizosphere of a salt tolerant Plant species, *Hordeum secalinum*	Tet*racycl*ine	Bcr/CflA family	Cardinale et al., 2015
*Alcaligenes*	*A. faecalis* ZD02	Monterey pine Agricultural soil, Japan	Streptomycin, Kanamycin, Penicillin, Amikacin, Cephalosporin, Isepamicin, Butirosin, Paromomycin, Gentamicin B, Neomycin, Ribostamycin, and Monobactam	aph6ID, aph33IB, aph3VIA, bl2BE, and sul1	Dwivedi et al., 2009; Ju et al., 2016
*Azotobacter*	*A. vinelandii strain* DSM85	Commercial Rhizosphere wheat	Chloramphenicol, Bacitracin, and Fluoroquinolone	bacA, mexF	Silini et al., 2012
*Acinetobacter*	*A. radioresistens*	Sweet potato rhizosphere	Chloramphenicol, Streptomycin, Kanamycin, Penicillin, and Tetracycline	ND	Yasmin et al., 2009
*Azospirillum*	*A. brasilense strain* Sp245	Island of Nicobar Digitaria rhizosphere	Chloramphenicol	MFP family, mdtA, mdtB, and mdtC	Wisniewski-Dye et al., 2011
*Agrobacterium*	*A. radiobacter* D14	Rhizosphere soil of the arsenic Hyperaccumulating plant *Pt. vittata*	Chloramphenicol, Tetracycline, Bleomycin, Bicyclomycin, Fosmidomycin, and Bacitracin	bacA, rarD, EmrB/QacA family, Bcr/CflA family	Wang et al., 2011
*Enterobacter*	*E. cloacae* strain 13,047	Marine oil-degrading bacterium Chili pepper seedlings Human cerebrospinal fluid	Tetracycline, Chloramphenicol, Aminoglycoside, Beta-lactamines, Glycylcycline, Macrolide, Cephalosporin, Sisomicin, Dibekacin, Penicillin, Tobramycin, Gentamicin, Netilmicin, Acriflavin, Carbapenem, Fluoroquinolone, Trimethoprim, and Sulfonamide	aac3IIA, aac6IB, acrA, ant2IA, bl2F, bl3IMP, bl1AMPC, bl3VIM, dfrA17, catB3, catA2, qnrA, sul1, sul2, and tetD	English et al., 2010; Ren et al., 2010
	*E. asburiae*	Rhizosphere of Quinoa	Tetracycline, Ampicillin		Mahdi et al., 2021a
*Gluconacetobacter*	*G. diazotrophicus* Pal5	Sugarcane N-fixing endophyte	Novobiocin	Bcr/CflA family, EmrB/QacA family, mdtA, mdtB, and mdtC	Bertalan et al., 2009
*Mesorhizobium*	*M. cicero*	Chickpea rhizospheric soil and root Nodules	Nalidixic acid, Penicillin, Chloramphenicol, and Erythromycin		Pandey et al., 2018
*Streptomyces*	*S. coelicolor*	Halophytic plants, *Salicornia europaea* L. Sorghum plants	Tetracycline, Chloramphenicol, Bleomycin, Fusidic acid, Tylosin, Daunorubicin, Bicyclomycin, Erythromycin, Tunicamycin, Colicin, Tetracenomycin C, Oxetanocin A, Bacitracin, Puromycin, Methyl viologen, Oleandomycin, Sulfonamide, and Vancomycin	cmlR, cmlE5, drrA, drrB, msrA, fusH, oxrA, pur8, smvA, tcmA, terD, tetR, tetM, tetV, tlrC, and ydgK	Hsiao and Kirby, 2008; O’Rourke et al., 2009
*Rhodococcus*	*R. erythropolis* P30	Red fescue Cd/Pb/Zn mine tailings	Vancomycin, Mupirocin, Daunorubicin, and Chloramphenicol	cmlE7, drrA, marC, Bcr/CflA family, and EmrB/QacA family	Sekine et al., 2006; Álvarez-López et al., 2016
*Planomicrobium*	*P. chinense*	Rhizosphere of sunflower	Chloramphenicol, Streptomycin, and Hygromycin B	ND	Khan et al., 2018
*Stenotrophomonas*	*S. maltophilia*	Rhizosphere of sugarcane	Kanamycin, Ampicillin, Tetracycline, Gentamycin, and Streptomycin	ND	Awais et al., 2017; Singh and Jha, 2017
*Klebsiella*	*K. pneumoniae*	Rhizosphere of sugarcane	Ampicillin, Kanamycin, Tetracycline, and Streptomycin	ND	Awais et al., 2017
	*K. terrigena*	Sweet potato rhizosphere	Chloramphenicol, Streptomycin, Kanamycin, Penicillin, and Tetracycline	ND	Yasmin et al., 2009

ND, Not determined.

Humans are exposed to resistant bacteria, antibiotics, or ARGs by several routes including contaminated crops, livestock, surface, and groundwater (Figure 3). These ARGs can disperse into the indigenous bacteria that colonize agricultural soils *via* spontaneous mutation or horizontal gene transfer. They can then be transmitted into edible plants, ingested by animals or humans, and eventually acquired by clinical pathogenic bacteria (Chen Q. et al., 2017). In 2010, Knapp et al. (2010) highlighted the significant accumulation of ARGs in agricultural soils sampled since 1940, reflecting the increasing use and application of antibiotics over the last decades, especially some tetracycline-coding ARGs which increased 15 times comparatively to the concentrations found in the 1970s (Knapp et al., 2010). This contamination might induce measurable impacts on the composition of microbial soil communities (Riber et al., 2014).

**Figure 4:**
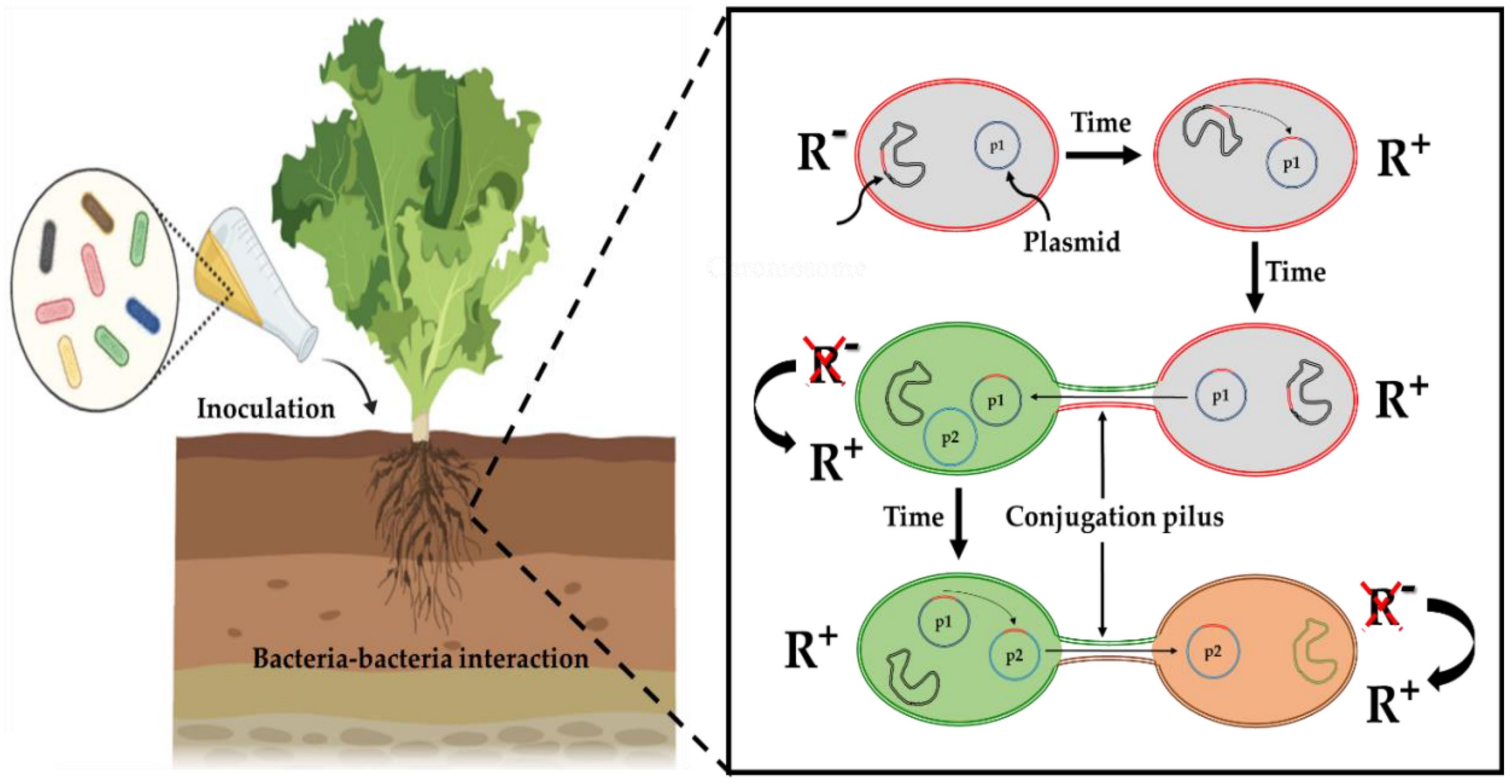
Extrinsic antibiotic resistance genes (ARGs) dissemination mechanisms upon PGPB application. ARGs (in red) appear in the chromosome and move by transposition to the plasmid (p1) within the same bacterium. Plasmids (p1) allow the transfer of ARGs between strains while plasmids (p2) allow transfer to distantly related strains. R^−^ indicates sensitivity and R^+^ indicates resistance.

Among the challenges to overcome for the commercial use of PGPB as biofertilizers, are their unstable characteristics, guarantee their survival and reinforce their competitiveness in soils in order to maintain a critical bacterial mass within the native microbiota (Cray et al., 2013). In addition, PGPB strains exhibiting intrinsic antibiotic resistance can serve as markers to assess the viability of inoculated bacteria in pot and field experiments (Kluepfel, 1993; Trivedi et al., 2004). Thus, isolation and screening of resistant PGPB are highly desired in order to ensure the wide adaptation of introduced bacteria to diverse habitats (Singh and Cameotra, 2013; Wani and Khan, 2013; Ma et al., 2016a). However, these explanations completely neglect the risk of biosafety related to the dissemination of multi-resistant PGP bacteria. For instance, cabbage inoculation with *Pseudomonas libanensis* and *Pseudomonas reactans* improved trace metal phytoremediation, therefore they were proposed as bioinoculants suitable to improve the phytoremediation of soils contaminated by metals in semiarid areas. These two strains were remarkably resistant to ampicillin, tetracycline, streptomycin, chloramphenicol, and penicillin (Ma et al., 2016b). Indeed, based on sequencing data, the vast majority of screened PGPB exhibit resistance to at least one antibiotic and/or harbor ARGs (Wellington et al., 2013; Kang et al., 2017). This may lead to immediate changes in the structure of bacterial community and eventually induce the dissemination of ARGs, if antibiotic pollutants are present in their environments. As shown in Figure 4, antibiotic-resistant bacteria can reach humans being by different routes mainly (i) consumption of plants/fruits that have been exposed to contaminated irrigation, manure, sludge, and slurry; (ii) using groundwater and surface water and residing animals (e.g., fish) containing resistant microbiota, and (iii) livestock that have been treated with veterinary antibiotics and exposed to resistant bacteria by the food chain. Antibiotic-resistant bacteria introduced into the soil as biofertilizers can subsequently be transported to surface water or groundwater and/or transfer their ARGs to inhabiting bacteria and be cycled within the environment thus enriching environmental reservoirs of ARGs (Topp et al., 2008; Figure 3). For instance, antibiotic-resistant bacteria colonizing plant tissues as endophytes, might transmit antibiotic resistance to consumers (Scaccia et al., 2021).

## Antibiotics as legacy pollutants

It has been pointed that by 2050 global annual mortality is projected to reach 10 million because of resistance to antimicrobials. Antibiotics are released into the environment *via* many pathways mainly clinical settings, human, and animal wastes, and through antibiotics supplemented food and fodder (Keswani et al., 2019).

As antibiotics have been widely used in agriculture as growth promoters in livestock, a large fraction of them are released into the environment creating a selective pressure for ARG dispersal in agricultural soils among others (Hung, 2020). Noteworthy, antibiotic resistance is evident in environments subjected to anthropogenic activities (Chen et al., 2013). This challenges the common point of view that ARGs solely occurs because of the contamination by antibiotics. In fact, it has recently been observed that other substances can also select for antibiotic resistance. This is becoming more commonly recognized, and regulators are beginning to monitor pathways and controlling the release of resistant-driving chemicals mainly antimicrobials, metals, and biocides (Singer et al., 2016). For example, metals and some persistent organic compounds (POCs) co-select for ARGs. More importantly, a study in Cuba revealed the presence of a high frequency of occurrence of ARGs along the Almendares River/estuary despite the minimal use of antibiotics in the country. However, high amounts of pollutants including metals, alongside other contaminants, were detected (Reid-Henry, 2008; Graham et al., 2011). Similarly, the abundance of ARGs was higher in soils amended with biosolids than in the surrounding agricultural soils without biosolids treatment. It was concluded that the application of biosolids also enhanced the emergence and spread of ARGs in the environment (Hung, 2020).

The co-selection of antibiotic resistance and metal or other pollutants can occur either by co-resistance or by cross-resistance. Co-resistance occurs when a phenotype is simultaneously selected with other genes located on the same genetic element. However, cross-resistance is developed when two pollutants (e.g., antibiotic and metal) have similar routes into the bacterial cell. In this case, when a resistance response is triggered, cell defense is effective against both metal and antibiotic contaminants. Under such contamination, selective pressures are likely to persist longer than pharmaceutical compounds because of the non-degradability of metals in the environment (Rodgers et al., 2019).

Furthermore, the presence of polycyclic aromatic hydrocarbons (PAHs) also induces shifts in the indigenous bacterial communities by increasing hydrocarbon-degrading bacteria (Zhang et al., 2010; de Menezes et al., 2012). Interestingly, many PAH-tolerant bacteria presented strong resistance to both metals and antibiotics (Máthé et al., 2012). In another study, several ARGs were found in PAH contaminated matrices (Chen B. et al., 2017). In fact, PAHs are pollutants (Liu et al., 2017) that can induce ARGs acquisition either by DNA transformation, or by triggering stress/repair systems. Although specific mechanisms are still unclear, a metagenomic profiling showed that PAH-contaminated soils harbor ARGs with approximately 15 times more abundance compared to those with low concentrations of contaminants (Chen B. et al., 2017). Finally, metals and PAHs are now established to exacerbate the emergence and dispersal of ARG in the environment and they can be used as model pollutants for further public-health risks related to these genetic pollutants. Nevertheless, ARGs co-selected by PAHs could be completely distinguished from those that have arisen from the use of antibiotics (Chen B. et al., 2017). Furthermore, soil analysis has also highlighted the impact of the non-clinical uses of antibiotics on ARGs spread as well as the impact of policy towards a more strict of their management in natural environments (Graham et al., 2016).

## Acquisition and dissemination of ARGs in soil microbiomes

Soil microbial communities were found to harbor cassettes of ARGs serving as a reservoir of genes potentially exchangeable with human bacterial pathogens (Forsberg et al., 2012). Additionally, the majority of soil-inhabiting bacteria that produce antibiotics [e.g., 2,4-diacetylphloroglucinol (DAPG), lipopeptides (LP), and phenazines] carry genes for the successful adaptation and self-defense against stressful conditions mostly located on the same gene clusters that confer resistance to other antibiotics (Tariq et al., 2022).

Noteworthy, the number of ARGs in plant associated-bacteria was found to be correlated with relatively higher levels of N-fertilizers (Forsberg et al., 2014). These ARGs are mostly carried by plasmids, transposable elements (Figure 4), and integrons (Supplementary Figure S2; Heuer et al., 2011; Gillings, 2017a). The antibiotic resistance of a bacterium can be natural (intrinsic). This type of resistance concerns all strains of a genus or a species and determines the wild-type resistance phenotype. Resistance can be carried by the chromosome and transmitted vertically to the progeny by cell division. Finally, antibiotic resistance can be acquired (extrinsic) by a greater or lesser proportion of the strains of a species and is variable over time. In this case, transmission is vertical or horizontal and occurs between different bacteria from different phyla *via* mobile genetic elements so-called the mobilome (Figure 4; Wellington et al., 2013; Ramakrishna et al., 2019). HGT occurs *via* transformation (integration of DNA in the environment), conjugation (sharing of plasmids between bacteria species), and transduction (bacteriophage mediated gene transfer; Vikesland et al., 2017). The concept of genetic exchange communities has been introduced by Jain et al. (2003) as a set of microorganisms that can exchange genes by HGT, without requiring physical proximity (Jain et al., 2003). Bacteria living within genetic exchange communities are susceptible to acquire ARGs by distinct bacterial species inhabiting separate environmental niches (Amarasiri et al., 2020).

In the 1980s, new genetic elements capable of acquiring or losing ARGs were described and named integrons (Stokes and Hall, 1989). Integrons are defined as genetic systems for capturing and expressing genes in the form of cassettes capable of being laterally transferred or excised by a site-specific recombination mechanism (Stokes et al., 2006). These cassettes are transcribed from a promoter (Pc) in the 5′- region and the expression of distal genes is reduced by the presence of upstream cassettes (Supplementary Figure S2). This system can incorporate open reading frames and convert them into functional genes expression systems. Interestingly, most gene cassettes encode ARGs and strains harboring integrons have been found in different environments. In 2001, Stokes et al. demonstrated the presence of several cassettes in samples from plant biomass, soil, sediments and water (Stokes et al., 2001). These integrons have a major role in the dissemination of antibiotic resistance in bacteria because they allow a very large number of combinations and therefore constitute an additional advantage for bacteria in the acquisition of ARGs (Ploy et al., 2005). The dispersal of ARGs in bacterial communities could also occur by lytic and temperate bacteriophages (Sharma et al., 2014).

The origin of acquired antibiotic resistance by PGPB could be the exposure of soils to anthropogenic activity or the production of antibiotics by antibiotics-producing bacteria inhabiting the soil.

Class 1 integrons were detected in crop associated bacteria mainly the β-proteobacterial class. This arguably creates a major conduit for the frequent influx of new mobile genes into bacteria closely associated with animals and humans at a scale whose magnitude remains to be explored (Stokes et al., 2006).

Many ARGs are responsible for intrinsic resistance to different antibiotics, including aminoglycosides, β-lactams, and fluoroquinolones. Intrinsic resistance in bacteria appeals mainly to the changes in cell wall permeability and activation of efflux pumps. Intrinsic resistance is a natural phenomenon adopted by many environmental bacteria especially Gram-negative ones (Ramakrishna et al., 2019). Moreover, some bacteria harbor genes called “cryptic genes” which are ARGs with very low expression levels. However, their expression could increase under binding conditions. This has been described in *Acinetobacter* (Zhang and Feng, 2016) and could be present in PGPB given their multiple environmental constraints.

## The major mechanisms of action of antibiotic resistance genes

The main modes of action of ARGs are: Inhibition of uptake, modification of target, inactivation of the antibiotic, and activation of efflux pump (Supplementary Figure S3; Ramakrishna et al., 2019). For instance, *Pseudomonas aeruginosa* often isolated from agricultural soil samples and characterized as a PGPB (Table 1), is a multi-drug resistant opportunistic pathogen harboring chromosomal and plasmid ARGs. Antibiotic resistance modes within this bacterium involve upregulation of β-lactamases and efflux pumps, alteration in membrane composition and chemical alteration of antibiotics (aminoglycosides; Lister et al., 2009; Poole, 2011). The antibiotic can be degraded or modified chemically by phosphorylation, nucleotidylation, acetylation, ADP-ribosylation, mono-oxygenation, or glycosylation (Vasala et al., 2020).

The high frequency of resistant bacteria is due to the high plasticity of the bacterial genome. Chromosomal mutations may occur or more frequently the acquisition of mobile genetic elements carrying ARGs found at high frequency in the bacterial cell. The acquisition of several ARGs by a bacterium results in resistance to many classes of antibiotics, usually called multidrug resistance (Wellington et al., 2013).

Many PGPB and biocontrol bacterial inoculants, especially *Pseudomonas* and *Bacillus* strains (Steil et al., 2003; Santoyo et al., 2012), are resistant to more than one antibiotic and/or harbor at least one ARGs (Wellington et al., 2013; Table 1). For instance, Fahsi et al. (2020) isolated 13 phosphate solubilizing bacteria (PSB), and checked their antibiotic resistance against kanamycin, streptomycin, tetracycline, ampicillin, chloramphenicol, and spectinomycin. Out of 13 PSB, 11 were able to resist, at least, one antibiotic (Fahsi et al., 2020). Similarly, *Serratia rubidaea* ED1 strain, an opportunistic pathogenic bacterium, isolated from quinoa roots endosphere was found resistant to seven antibiotics (Mahdi et al., 2021b). In addition, out of 11 PSB isolated from quinoa rhizosphere, five strains showed resistance to at least two antibiotics (Mahdi et al., 2021a). More recently, antibiotic resistance mechanisms were studied in biofertilizer samples following application into the soils, these include mainly antibiotic deactivation (48.7%), efflux pumping (25.2%), cellular protection (18.0%), and other unknown mechanisms (8.1%; Yang et al., 2022). In North America, streptomycin sulfate has been used to control heavy outbreaks of cultures. However, because of the risk of enhancing the rise and dispersal of antibiotic resistance in nontarget bacteria, European authorities have banned its use in Europe. Since then, ecofriendly alternatives have been eagerly searched for, and two biocontrol agents based on *Bacillus subtilis* were registered for blight control in Europe: Serenade® based on strain QST713 and Biopro® based on strain BD170 (Broggini et al., 2005). However, this species is susceptible to harbor antibiotic resistance genes in their genetic material (Table 1).

Based on a phylogenetic analysis, it is probable that the mobile elements hosting ARGs originates mainly from Firmicutes and Bacteroidetes (McHardy et al., 2007; Sommer et al., 2010). Hence, the introduction of these PGPB into soils can supply the ARGs reservoirs as donors and worsen the spreading risks of these genes in the soil microflora. The large-scale use of bacterial biofertilizers is not without consequences as they could serve as acceptors for ARGs in the soil and move more easily to plants with pili. Ultimately, the resistance of PGPB to antibiotics becomes a blatant paradox in biofertilization (Kang et al., 2017).

## Diversity of ARGs in plant-associated bacteria

Many studies have focused on the detection and quantitation of ARGs in agricultural soils, uncovering resistance mechanisms, and identifying novel enzymes responsible for the resistance of bacteria to antibiotics. As for those inhabiting soils, up to 177 ARGs conferring resistance to aminoglycosides, chloramphenicol, and β-lactams were reported in pristine Antarctic soils. It has been suggested that the vast majority of ARGs most likely represent functional historical genes encoding for resistance to natural antibiotics (Van Goethem et al., 2018).

Antibiotic resistance gene diversity and abundance are strongly affected by agricultural practices (Wepking et al., 2017). However, soil pH, the organic content, and the history of agricultural soil management have been documented as important drivers influencing the abundance and the fate of ARGs (Čermák et al., 2008; Popowska et al., 2012).

In a metagenomic study performed on Chinese soils to which manure was applied, ARGs conferring resistance against tetracycline, minocycline, streptomycin, kanamycin, gentamycin, amikacin, rifampicin, and chloramphenicol accounted for about 70% of the total ARGs detected. More than 60% of identified genes presented low similarity (less than 60%) compared to the sequences of their closest proteins at the amino acid level (Su et al., 2014). Noteworthy, among the studied ARGs, the average abundance of *tetO* and *ampC* genes in the soil treated with manure was 3.3 and 421% higher comparatively to manure free soil, respectively (Wepking et al., 2017).

A broad-spectrum diversity of ARGs such as *tetA, sul1, blaOXA2*, *blaCTX-M*, and *qnrS* has been shown by Nõlvak et al., (2016) in soils subjected to treatment by animal manure. The rate of tetracycline resistance encoding genes namely *tetM, tetO*, and *tetW*, was significantly greater in manure-treated soils (Xiong et al., 2018). This was explained by an increasing number of ARGs resulted from antibiotic-resistant bacteria already present in manure. This led to a selective pressure of some antibiotic-resistant bacteria to dominate the microbial community.

As for biofertilizers application, a recent study assessed ARGs and mobile genetic elements (MGEs) harbored by biofertilizer and soil samples using high-throughput quantitative polymerization chain reaction (HT-qPCR). Fluctuation in specific ARGs [*QnrB4*, *vanC2_vanC3*, *copA*, *sugE*, *mepA*, *tetD*, and t*etA(P)*] and MGEs [*IS26* (MGEs) and *IS6100* (MGEs)] were noticed following biofertilizer application. However, the overall relative abundance of ARGs was no significant at the applied dose but the authors suggested that biofertilizers application should be evaluated over time as resistance transfer is time dependent (Yang et al., 2022). Similarly, using genomic analysis, Nordstedt and Jones (2021) identified genes putatively involved in antibiotic resistance in the PGPR *Serratia plymuthica* MBSA-MJ1 namely chloramphenicol acetyltransferase (*cat*) gene providing resistance to chloramphenicol and six copies of the efflux pump responsible for tetracycline resistance (*stp*; Nordstedt and Jones, 2021). Other identified genes include those encoding for resistance to fosmidomycin (*fsr*), bicyclomycin (*bcr*), polymyxin (*arnABCD*), and β-lactam multi-drug resistance (*ampC*, *ampD, and ampR*). In some soils, plasmids harbor *bac* gene conferring resistance to bacitracin in *Bacilli* strains. This gene is acquired by HGT and is mandatory for their survival in most soils (Ramakrishna et al., 2019). Nevertheless, the diversity and novelty of ARGs are likely to pose risks to humans as they can transfer the ARGs to human pathogenic bacteria (Su et al., 2014).

## Relevant diagnostic tools for detecting lateral gene transfers of ARGs

Horizontal gene transfer (HGT) is well documented as an important mechanism in bacterial evolution (Popa and Dagan, 2011). It provides rapid access to novel functions, allowing traits such as virulence, antibiotic resistance, and xenobiotic metabolism to spread through the human microbiome as well as plant-associated microbiome (Smillie et al., 2011). Yet, current HGT detection techniques [e.g., *LatTrans* (Hallett and Lagergren, 2001)*, RIATA-HGT* (Than et al., 2008), or *HGT-Detection* (Boc et al., 2010)], are hardly usable for building networks having more than 100 leaves. To circumvent this problem, some reports implemented a fast distance-based method for the inference of HGT networks comprising thousands of species. Then, they developed an accurate probabilistic algorithm for detecting partial HGTs (Denamur et al., 2000). In a study involving a network of 10,770 unique genes found in 2,235 full bacterial genomes, it has been shown that HGT networks have been shaped principally by ecology rather than geography or phylogeny, with most gene exchange occurring between isolates from ecologically similar, but geographically separated environments (Smillie et al., 2011).

Many diagnostic methods are being used to detect ARGs from different environments. In this section, we provide an outline of some current and emerging tools for diagnostics and fast detection of ARGs (Kaprou et al., 2021; Table 2).

**Table 2:** Summarizing table of relevant diagnostic tools for detecting lateral gene transfers of ARGs.

Conventional methods		Non-conventional methods		Microfluidics
Molecular techniques	PCR-based methods	Genome sequencing and metagenomics	Whole genome sequencing (WGS)	Quartz-crystal microbalance (QCM)
	Isothermal amplification methods		Pyrosequencing	
	DNA microarrays		Short and long read combination	
			Nanopore sequencing	
		MALDI-TOF mass spectrometry		

### Conventional ARGs diagnostic methods

The conventional methods relying on molecular-based technologies provide fast and sensitive detection of ARGs and have extended the availability of huge ARGs targets in various databases (Waseem et al., 2019). In the following sub-section, nucleic acid amplification-based techniques such as PCR, isothermal techniques, and DNA microarrays are discussed.

#### PCR-based methods

PCR is the most used method for the detection of ARGs (Chisholm et al., 2001; Seedy et al., 2017). Recently, quantitative (Böckelmann et al., 2009), real-time (Mackay, 2004), digital (Bogožalec Košir et al., 2020), and multiplex PCRs (Jousset et al., 2019) have been further incorporated for genetic assays. Moreover, technological advances made for the next-generation sequencing (NGS) and whole genome sequencing (WGS) have greatly influenced the ARGs targets and paved the way to the high throughput quantitative PCR (HT-qPCR). This is faster, suitable for the simultaneous exploration of many ARGs (Waseem et al., 2019), and applicable for ARGs originating from different sample types including soils (Waseem et al., 2020). For instance, using HT-qPCR, Wang et al. (2014) explored and profiled bacterial ARGs from urban park soils (Wang et al., 2014). Indeed, this technology has been successfully employed using various sample types such as water, animal feces, and soil (Xu et al., 2019).

#### Isothermal amplification methods

The isothermal DNA amplification is advantageous over conventional PCRs because it does not require thermocycling. In addition, it is faster, more sensitive, and more specific (Zanoli and Spoto, 2013; Zou et al., 2020). This method includes several techniques, such as strand displacement amplification (SDA), nucleic acid sequence-based amplification (NASBA), transcription mediated amplification (TMA), rolling circle amplification (RCA), loop-mediated isothermal amplification (LAMP), recombinase polymerase amplification (RPA), and helicase-dependent amplification (HDA; Gill and Ghaemi, 2008). Recently, it was that LAMP and RPA have great potential for point-of-need diagnostics used in low resource settings (Zou et al., 2020). *In vitro* diagnostic products based on PCR and isothermal nucleic acid amplification technology (NAAT) are commercialized (Morin et al., 2018). The automation of each processes (from DNA extraction to gene detection) coupled with data analysis software tools have resulted in accurate integrated platforms (Cantera et al., 2019).

#### DNA microarrays

A DNA microarray allows the detection of genes in a target organism when compared to a reference genome or strain. Using fluorescently labeled PCR sequences (DNA chips), simultaneous detection of ARGs among *Staphylococcus* clinical isolates was achieved (Zhu et al., 2007). Recently, a rapid cartridge based, melting curve assay to detect pyrazinamide resistant *Mycobacterium tuberculosis* was proposed (Havlicek et al., 2019).

### Non-conventional ARGs diagnostic methods

In this section, some of the most promising non-conventional methods for ARGs detection are described. They include sequencing, matrix-assisted laser desorption/ionization, time-of-flight mass spectrometry (MALDI-TOF MS), and Fourier transform infrared (FTIR). spectroscopy.

#### Genome sequencing and metagenomics

Numerous methods, tools, and databases have been reported in recent years for the detection of ARGs from WGS and WMS data (Köser et al., 2014; Oniciuc et al., 2018). These evolving technologies provide rapid and sensitive determination of resistances in both uncultivable and cultivable bacteria (Kaprou et al., 2021).

##### Pyrosequencing

The identification of ARGs using pyrosequencing was developed by Amoako et al. (2012). It served as a tool for the detection of clinical drug-resistant *Mycobacterium tuberculosis*. Reliable and robust detection of resistance-associated mutations in *M. tuberculosis* isolates was achieved with very high specificity (96–100%; Ajbani et al., 2014). This method was also efficient to rapidly detect resistances to kanamycin, fluoroquinolones, rifampicin, and capreomycin in *M. tuberculosis* clinical strains (Govindappa et al., 2016). This assay was considered as a fast and effective method for the detection of mutations associated with drug resistance in *M. tuberculosis* clinical isolates (Govindappa et al., 2016).

##### Whole genome sequencing

Several studies have highlighted that WGS is a powerful tool for ARGs surveillance (Alghoribi et al., 2018; Hendriksen et al., 2019; Argimón et al., 2020). Vélez et al. (2017) used WGS for the prediction and determination of ARGs occurrence in *Streptococcus uberis* and *Streptococcus dysgalactiae* strains isolated from dairy cows. This showed the association between several unique ARGs sequences and phenotypic resistances (Vélez et al., 2017). Similarly, Zhao et al. (2016) identified ARGs in *Campylobacter* and checked their correlation to phenotypes by employing *in vitro* antimicrobial susceptibility testing and WGS (Zhao et al., 2016). A strong positive correlation (99.2%) was seen between resistance patterns and genotypes. These findings indicate that WGS is a reliable resistance indicator.

##### Short and long read combination

As mentioned above, plasmids can spread ARGs among bacteria. However, their reconstruction from short-read WGS data is difficult. Short and long read WGS sequencing was used to characterize and locate ARGs situated on plasmids (Berbers et al., 2020). In fact, it is of crucial importance to establish ARGs location, especially when they are in mobile elements. Biosafety assessment of ARGs transfer was feasible by overcoming the issues of plasmid assembly when employing the combination of long and short read sequencing (Berbers et al., 2020).

##### Nanopore sequencing

Nanopore sequencing has been used for the identification of ARGs in Enteroaggregative *E. coli* (EAEC) as well as their position and structure (Greig et al., 2018). Analysis of WGS data enabled easier identification of ARGs and revealed the combination of many ARGs determinants located on the same mobile element. These findings provided a profound understanding of the transmission of co-located ARGs determinants in EAEC (Greig et al., 2018). Schmidt et al. (2017) showed that nanopore-based metagenomic sequencing successfully identified acquired ARGs directly from urine samples (Schmidt et al., 2017). The Oxford Nanopore MinION long read DNA sequencing device was used to detect ARGs, assess their taxonomic origin, and decode their genetic organization and possible correlation with mobilization markers. Therefore, targeted interventions can be established to alleviate the risks of ARGs transfer among sites and, thus, improve biosecurity practices in hospitals and other environments (Kamathewatta et al., 2019). More recently, MinION nanopore sequencing was employed for rapid identification of pathogens, plasmids and ARGs in bacterial DNA (Taxt et al., 2020). It accurately predicted the multilocus sequence type and identified ARGs profiles (Tan et al., 2020). Similarly, the ultra-long read nanopore sequencing technology was employed for ARGs detection in *Mannheimia haemolytica* (Lim et al., 2019).

#### MALDI-TOF mass spectrometry

Matrix-assisted laser desorption/ionization time-of-flight mass spectrometry (MAL-DITOF MS) can also be employed for the detection of ARGs (Justesen et al., 2018; Paul et al., 2018; Liu et al., 2019). MALDI-TOF MS relies on profiling proteins from bacterial cell extracts creating a spectral fingerprint that discriminates bacteria at subspecies level (Angeletti, 2017; Welker and van Belkum, 2019). It has also been used for the identification of antibiotic resistance mechanisms (Hoyos-Mallecot et al., 2014). However, the large size and high cost of MAL-DITOF MS systems substantially restricts its implementation for these purposes (Welker and van Belkum, 2019). Additionally, it requires purification, cultivation as well as sample preparation that are not suitable for studying mixed samples (Wang et al., 2018). This being said, MALDI Biotyper and VITEK MS are two commercially available MALDI-TOF MS systems (Marko Daniel et al., 2012; Martiny et al., 2012; Byun et al., 2018).

#### Microfluidic methods

Quartz-Crystal Microbalance (QCM) is a microfluidic technology made of a physical nanogram-sensitive device with a piezoelectric sensor. QCM facilitates the real-time, rapid, on-site detection of antibiotic resistant bacteria (Bragazzi et al., 2015). This approach’s high sensitivity, accuracy, and dynamics allowed for the detection of ARGs using a magnesium zinc oxide nanostructure-modified quartz crystal microbalance (MZO_nano_-QCM) biosensor (Reyes et al., 2017b). QCM is advantageous because of its low cost, low demand in sample volume, and rapidity (Reyes et al., 2017a).

## Some mitigation strategies to reduce ARGs dissemination

Although reassessment of the biosafety threat of PGPB formulations is under review in some countries, though mainly in North America and Europe (Abhilash et al., 2016), preventive actions must be taken to play down the conjunction of factors that worsen the emergence, evolution, and dispersal of antibiotic resistance within arable lands. The possible ways to cope with the issue of antibiotic-resistant and/or pathogenic PGPB include: (1) polyphasic characterization of bioinoculants at the species and strain level, (2) developing risk assessment groups for biofertilizers recommendation (Tariq et al., 2022), (3) prioritizing the studies on the resistance profiles of PGPB and the potential risks of disseminating ARGs in PGPB before field experiments and large-scale applications, (4) favoring antibiotic sensitive PGPB strains and those with fewer ARGs, (5) excluding multidrug-resistant strains, (6) inoculating with PGPB by spraying the aerial parts of plants or by soaking seeds, (7) prioritizing the use of promoting and/or biocontrol metabolites produced by PGPB, (8) reducing reliance on genetically engineered PGPB with ARGs biomarkers, (9) excluding bacterial species known to be pathogenic to humans, (10) avoiding PGPB with special ARGs genotypes that do not occur in the human microbiome (Kang et al., 2017; Ramakrishna et al., 2019), (11) using biochar as a soil amendment to reduce the abundance of resistance genes such as those conferring resistance to tetracyclines (*tetC, tetG, tetW, and tetX*) and sulfonamides (*sulI and sulII*) in soil and plant tissues (Ye et al., 2016; Duan et al., 2017), (12) genome mining PGPB strains for more precise characterization and identification of highly effective biofertilizers and potential harmful features, and (13) establishing standard criteria, regulations, and quality control guidelines for premarket biosafety assessment and post market monitoring of commercial biofertilizers. Finally, scientists should also identify which ARGs to survey for risk assessment in bacteria-based inoculants to distinguish those that are most relevant to human health.

## Putative regulatory framework

As the biofertilizers market still lags behind synthetic fertilizers, greater attention should be played to regulatory aspects to share responsibilities in overcoming the challenges and offering high-quality products. Among the major constraints, antibiotic resistance across agroecosystems is urgent to combat with a target on prevention and remediation prospects. Commercialization should also require strict reference systems to demonstrate their effectiveness and potential associated risks. In developing countries, the spread of this public health scourge is due to (i) the lack of surveillance of resistance occurrence, (ii) the poor quality of utilized antibiotics, (iii) the clinical and agricultural excessive/misuse, and (iv) the improper and easy disposal of antibiotics and pharmaceutical wastes. However, these factors differ from developing to developed countries. Overall, there is still a need to improve and update the regulatory aspects for antibiotic use especially in agriculture (Chokshi et al., 2019). In the following section, we sought to contribute to set a putative regulatory framework for the use bacterial-based biofertilizers to help guarantee their biosafety, tackle the antibiotic resistance issue, and ensure environmental and public health protection.

Isolated and screened strains should be taxonomically characterized using a polyphasic approach appealing to phenotypic (morphological, physiological, and biochemical), chemotaxonomic (peptidoglycans, fatty acids, proteins, sugars…), and molecular aspects (Barcoding; Supplementary Figure S4; Keswani et al., 2019). In addition to *in silico* taxonomic profiling, it is mandatory to compare the potential strains physiologically and morphologically with closely related ones for phylogenetic delineation (Stackebrandt et al., 2002; Trujillo, 2011).

Nowadays, whole genome sequencing is a more comprehensive strategy to taxonomically and functionally identify the strain of interest and to assign it with its closest relatives. Subsequently, depending on their biosafety level, candidate strains should be classified within risk group (BSL-1, 2, 3, and 4). Bacteria falling in BSL-1 class are more likely to be null of biosafety risk. This implies further experiments such as formulation and testing. However, strains falling in the BLS-2 class of higher are more likely to be risky with potential negative impact. In this case, pathogenesis investigation and characterization at the strain level should be carried out. When it is a pathogenic species, it should be excluded from downstream experiments unless alternatively used, under confinement conditions in the laboratory, to optimize the production of metabolites and compounds of interests (Supplementary Figure S4).

To promote the acceptance of biofertilizers by the users, the quality of a bioinoculant must refer not only to the specification of the strain, efficacy, and preservation etc. but also to its antibiotic resistance profile and potential pathogenesis for human. This information can be reflected in the labelling requirements (Malusá and Vassilev, 2014).

Controlling biofertilizers is essential to ensure their compliance with standards, product safety, and efficacy. Poor quality products can be expected when the regulatory setup is not well established, leading to poor performance in the field or potential health risks to users. The lack of effective regulation of biofertilizers in most countries is one of the major factors to their low availability and adoption. Afterwards, periodic monitoring of commercialized biofertilizers is important to ensure product quality and safely throughout the marketing chain (Pandey and Chandra, 2016).

## Conclusions and prospects

To mitigate the risks posed by the continued emergence of antibiotic resistance, behavioral changes and practical strategies should be considered by all the stakeholders. In agriculture, the trend of PGPB, which is claimed to be sustainable and environmentally friendly, could be a significant threat and cause large-scale dissemination of multi-drug resistant bacteria and/or ARGs in different environments including human beings. The future development of more effective bacteria-based biofertilizers should consider environmental issues as there is enough evidence supporting the hypothesis that antibiotic-resistant biofertilizers could contribute substantially to the global antibiotic resistance crisis. Additionally, understanding the underpinning mechanisms is necessary to develop effective approaches in the battle against the spread of antibiotic resistance. Ultimately, to tackle this issue at its source, better formulated strategies are needed based on agricultural, environmental, and public health aspects.

The need for knowledge of the antibiotic resistome in plant agricultural systems and especially those in which biofertilizers are applied is critically important because we need to understand whether their application to the soil has the potential to select ARGs that could impact human health. As this has the potential to impact human health, and current implications for the use in agriculture, commercial biofertilizers should be well characterized with respect to their ability to transfer ARGs to other microorganisms. Detecting organisms harboring ARGs and their identification is important for risk assessments with co-inhabiting antibiotic-resistant communities. If ARGs of importance in clinical medicine are identified in the resistome of bioinoculants and/or inoculated plants, it is critical to determine whether their frequency and/or bacterial host range changes based on strain exposure. Finally, more in depth studies should address the genetic behavior, the bacterial diversity, and the activity of PGPB harboring ARGs with regards to indigenous communities.

## Author contributions

IM and NF reviewed the literature and wrote the original draft of the manuscript. MH reviewed and corrected the manuscript. MS revised the manuscript. IM, MH and MS designed and conceived the work. All authors contributed to the article and approved the submitted version.

## Funding

This work was supported by funding from the OCP Group (Projects AS-78 and AS-85, awarded to MH) and Mohammed VI Polytechnic University (UM6P, awarded to MS), which are greatly acknowledged.

## Conflict of interest

The authors declare that the research was conducted in the absence of any commercial or financial relationships that could be construed as a potential conflict of interest.

## Publisher’s note

All claims expressed in this article are solely those of the authors and do not necessarily represent those of their affiliated organizations, or those of the publisher, the editors and the reviewers. Any product that may be evaluated in this article, or claim that may be made by its manufacturer, is not guaranteed or endorsed by the publisher.
