# Supplementary material for: Antibiotic resistance in plant growth promoting bacteria: A comprehensive review and future perspectives to mitigate potential gene invasion risks
Source: Front Microbiol. 2022 Sep 20;13:999988. doi: 10.3389/fmicb.2022.999988 (PMC9530320; doi:10.3389/fmicb.2022.999988)
Supplement: Supplementary file 1 [file Data_Sheet_1.docx]

**Antibiotic Resistance in Plant Growth Promoting Bacteria: A comprehensive Review and Future Perspectives to Mitigate Potential Gene Invasion Risks**

***Supplementary Material***


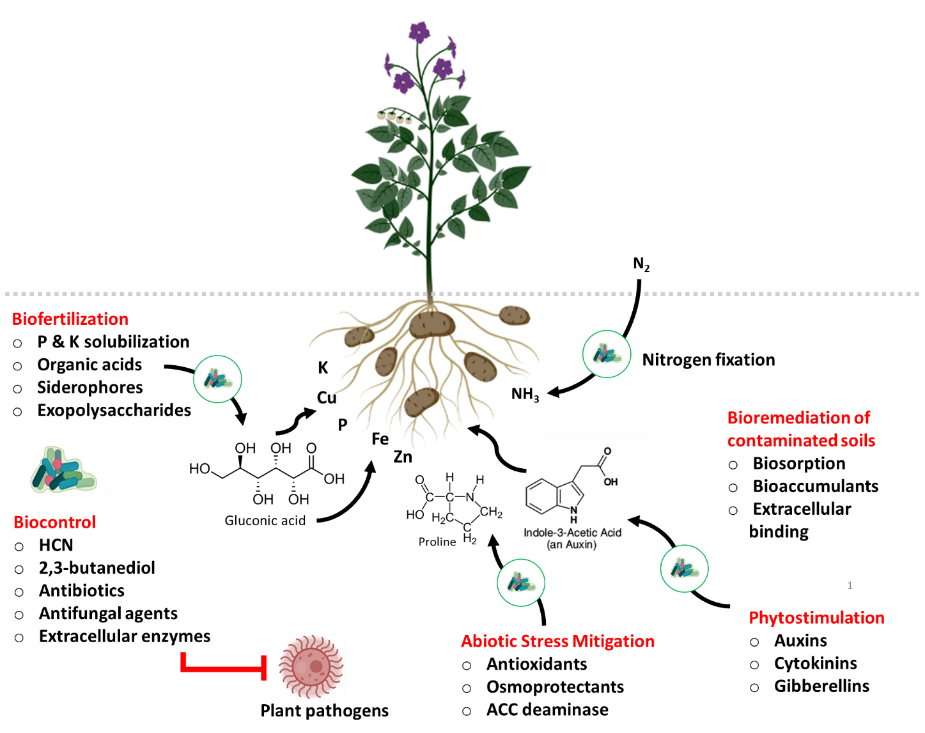


**Figure S1.** Schematic representation of host plant – PGPB interactions and different mechanisms used by PGPB to boost nutrients acquisition, alleviate abiotic stress, and inhibit the phytopathogens.


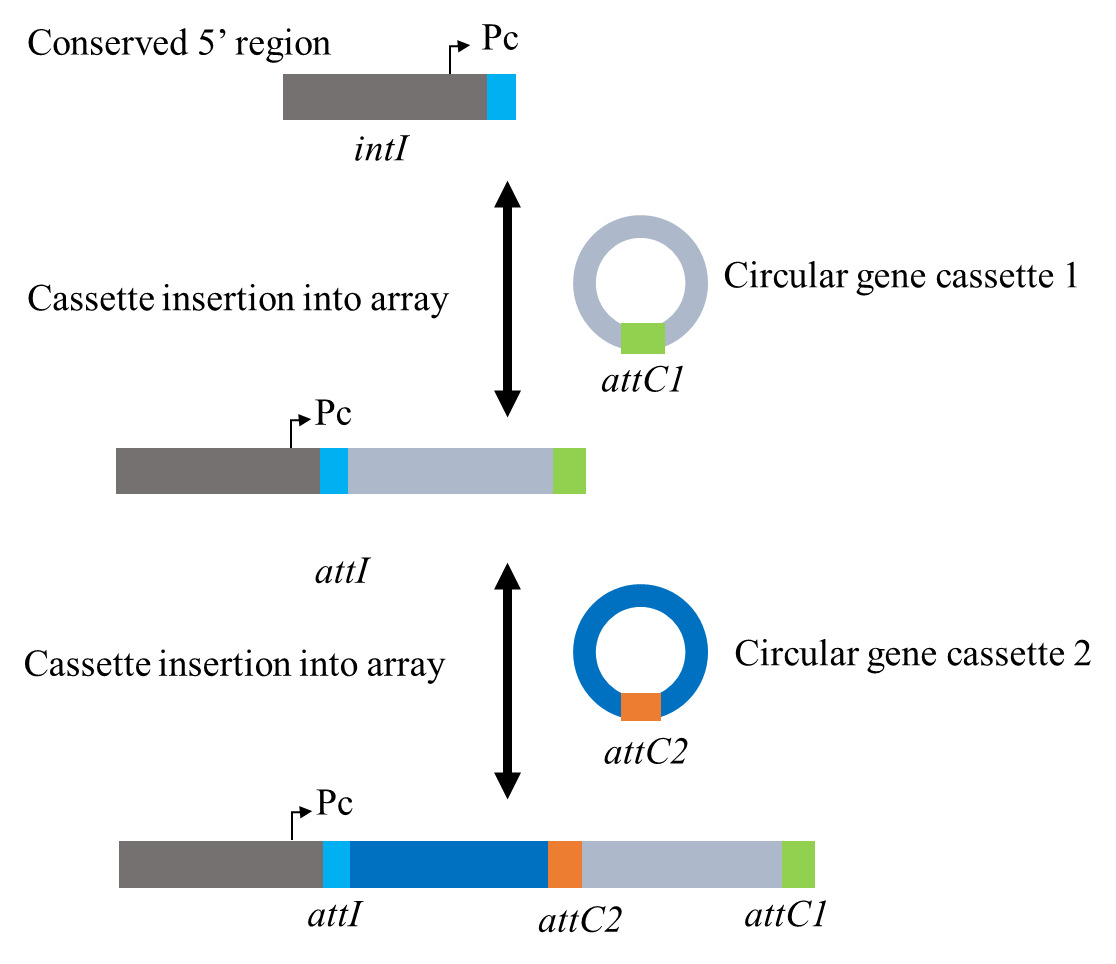


**Figure S2.** Antibiotic resistance gene acquisition by integrons. Pc: Promotor, *intI*: Integrase gene, *attI*: Integrase recombination site, *attC*: Cassette’s recombination site. The integration of the cassettes preferably takes place at the *attI* recombination site located in the 5′ region. Different cassettes can be integrated, each with a unique *attC* site.


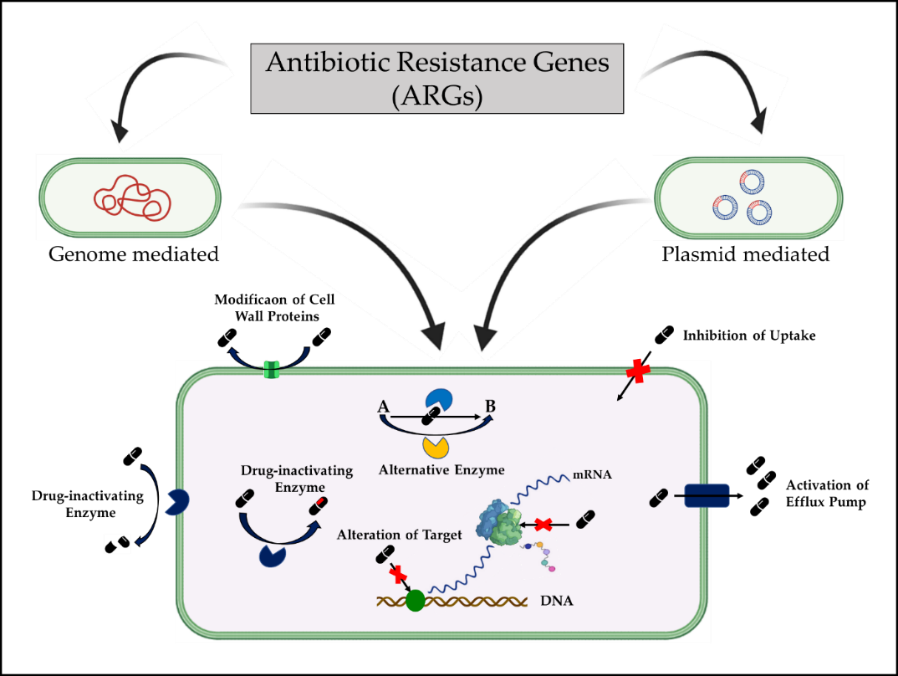


**Figure S3.** Bacterial targets and mechanisms of action of intrinsic antibiotic resistance genes (ARGs) of plant growth promoting bacteria (PGPB). ARGs could evolve either from chromosome or plasmid. The main mechanisms of antibiotic resistance are impermeability, enzymatic inactivation, modification of the antibiotic's target, and active efflux.


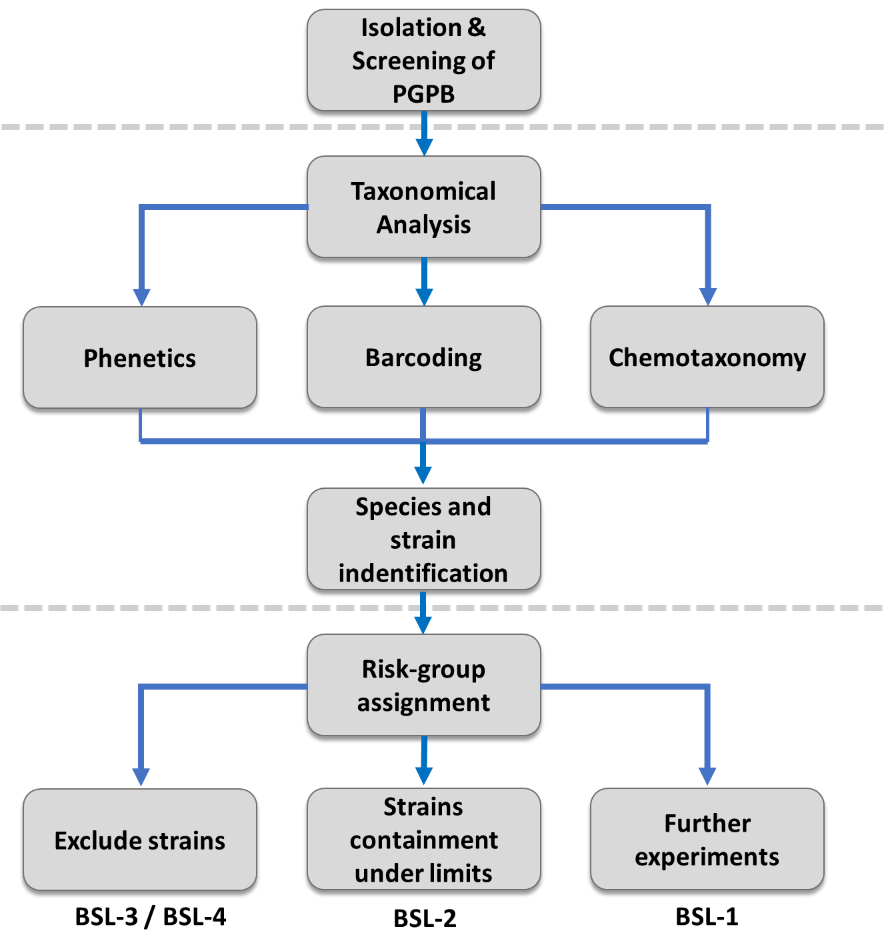


**Figure S4.** Characterization steps of [biofertilizers](https://www.sciencedirect.com/topics/earth-and-planetary-sciences/plant-growth-promoting-rhizobacteria) using polyphasic approach ([Keswani et al., 2019](#_ENREF_84)).
